# Temporal transcriptome highlights the involvement of cytokine/JAK/STAT3 signaling pathway in the osteoinduction of BMSCs

**DOI:** 10.1186/s13018-023-03767-9

**Published:** 2023-04-10

**Authors:** Xiao Ru, Peian Cai, Manli Tan, Li Zheng, Zhenhui Lu, Jinmin Zhao

**Affiliations:** 1 Guangxi Engineering Center in Biomedical Material for Tissue and Organ Regeneration, Guangxi Medical University, Nanning, 530021 China; 2 Collaborative Innovation Centre of Regenerative Medicine and Medical BioResource Development and Application Co-Constructed by the Province and Ministry, Guangxi Medical University, Nanning, 530021 China; 3 Life Science Institute, Guangxi Medical University, Nanning, 530021 China

**Keywords:** Temporal transcriptome, Bone marrow mesenchymal stem cells, Osteogenesis, Angiogenesis, Cytokine/JAK/STAT3 signaling pathway

## Abstract

**Background:**

Mesenchymal stem cells (MSCs)-based therapy offers an effective strategy for bone regeneration to solve the clinical orthopedic problems. However, the transcriptional regulation of multiple transitional stages of continuous osteogenesis from MSCs has not been fully characterized.

**Methods:**

Bone marrow mesenchymal stem cells (BMSCs) stimulated with osteogenic induction media were utilized to construct the in vitro osteogenic differentiation model. BMSCs were harvested after induction for 0, 7, 14 and 21 days, respectively, to perform the mRNA-sequencing (mRNA-Seq). The transcription factor networks and common molecules during the osteogenesis were revealed by using the temporal transcriptome. Further verification was performed by the quantitative real-time polymerase chain reaction (qRT-PCR), immunofluorescence and Western blotting.

**Results:**

It showed that BMSCs could differentiate into osteogenic, and crucial regulator in the MAPK signaling pathway, the PPAR signaling pathway, the Toll-like receptor signaling and the Cytokine/JAK/STAT signaling pathway. PPI protein interaction analysis also suggested that three cytokines are involved in osteogenic differentiation as core genes, including leukemia inhibitory factor (LIF), interleukin-6 (IL6) and colony-stimulating factor 3 (CSF3). The osteogenic process was negatively affected by the inhibition of JAK/STAT3 signaling pathway.

**Conclusions:**

This work might provide new insights in the crucial features of the transcriptional regulation during the osteogenesis, as well as offer important clues about the activity and regulation of the relatively long-activated Cytokine/JAK/STAT3 signaling pathway in osteoinduction of BMSCs.

## Introduction

A wide range of bone defects and nonunion caused by trauma, tumor metastasis and chronic osteomyelitis are common orthopedic challenges in clinics, leading to a serious decline in people's quality of life and greatly increasing the social burden [1]. Mesenchymal stem cells (MSCs) are a kind of multipotent system cells that exist in many organs, including umbilical cord blood, bone marrow, fat and synovium [2]. Moreover, bone marrow mesenchymal stem cells (BMSCs) are precursors of osteoblasts and directly participate in the growth and remodeling process of the bone by regulating the differentiation of osteoblasts. Because the osteogenic differentiation of BMSCs is affected by induction time, clarifying the detailed mechanisms of osteogenic differentiation of BMSCs would be contributed to developing the new strategies for bone regeneration. Bone regeneration begins with acute inflammatory response and then regeneration or degeneration phase may occur because of the cross-talk between immune cells [3]. The initiation of inflammation leads to the recruitment of immune cells and stem cells. Complete deficiency of TNF-α and IL6 had been reported to delay skeletogenic MSCs differentiation [4]. IL6, as one of immuno-inflammatory response factors related to bone diseases, could potentiate osteogenic effects of bone morphogenetic protein-2 (BMP-2) via BMPR1A-mediated BMP/SMAD pathway [5]. Hypoxia regulated angiopoiesis and osteogenesis in human osteoblasts by upregulating the expression level of IL6 [6]. Another IL6 family cytokine, leukemia inhibitory factor (LIF), is also involved in the formation of calcified cartilage and its vascularization for bone development [7].

STAT3 is a protein in the Janus kinase signal transducers and activators of transcription (JAK-STAT) signaling pathway which is commonly expressed and presents as an inactive form in the cytoplasm. In the activation of a variety of signaling, such as IL6 family cytokines and growth factors including colony-stimulating factor 3 (CSF3) and vascular endothelial growth factor (VEGF), it can be transiently and rapidly tyrosine phosphorylated [8, 9]. In the induction of Toll-like receptors (TLRs), IL6 was triggered and collaborated with its receptor (sIL6R) for osteogenesis by enhancing the phosphorylation of STAT3 [10]. Additionally, inhibition of JAK2/STAT3 signaling pathway led to suppression of VEGF expression [11]. Indicating the Cytokine/JAK/STAT3 signaling pathway may be participated in regulating the osteogenic and angiogenic induction of BMSCs.

MSCs cultured with osteogenic induction media is a feasible and noncancerous model for investigating the osteogenesis and bone regeneration which has been used in previous studies [12]. The temporal transcriptome which is a transcriptomic analysis of samples collected at different periods is able to identify similarity and diversity of each phase. In this study, the temporal transcriptome of osteogenic differentiation was investigated by inducing osteogenic differentiation of BMSCs with osteogenic induction media and performing the RNA-sequencing (RNA-Seq) after stimulation of 0, 7, 14 and 21 days. Significant molecular signatures at different stages of osteoinduction were identified by systematic computer analysis and their interaction with angiogenesis was also explored, which would offer reference for understanding transcription of osteogenesis of BMSCs in bone regeneration.

## Material and methods

### Cells extraction and induction

The primary BMSCs were extracted from new-born New Zealand rabbits according to our previous study with the permission of the ethics committee of Guangxi Medical University (Nanning, China) [13]. The new-born rabbits were killed with overdoes pentobarbital sodium. Subsequently, the bilateral femurs were collected and the bone marrow-MSCs (BMSCs) were harvested by flushing the femoral marrow cavities with alpha-modified Eagle's medium (α-MEM, Hyclone, USA) supplemented with 10% of fetal bovine serum (Gibco, USA) and streptomycin (0.1 mg/mL)/1% penicillin (100 U/mL) (Solarbio, Beijing, China). For osteogenic differentiation of BMSCs, cells at passage two were collected and induced with osteogenic induction medium (50 ug/mL of ascorbic acid, 10 nM of dexamethasone and 0.02 M of β-glycerol phosphate) [12].

### Alizarin red staining

In the induction of 0, 7, 14 and 21 days, respectively, cells were washed 3 times with phosphate-buffered solution (PBS). Followed by fixing with paraformaldehyde (4%) for half an hour and rinsing 3 times with PBS again, 0.2% of alizarin red (Solarbio, Beijing, China) was added for staining for 1 h. A microscope (Olympus, Japan) was utilized for observation and photography.

### Alkaline phosphatase (ALP) activity

At day 0, 7, 14 and 21 of incubation by osteogenic induction medium, cells were collected and lysed with lysis buffer (Beyotime, Beijing, China) for 30 min. Subsequently, the lysate was collected by centrifugation and the protein concentration was assessed by a BCA kit (Beyotime, Shanghai, China). The ALP activity was detected by an ALP detection kit (Beyotime Biotechnology, Shanghai, China) at a wavelength of 405 nm by using a microplate reader (Thermo Fisher Scientific, USA).

### Alp staining

Cells were fixed with 4% of paraformaldehyde. After washing with PBS, the ALP staining was carried out by an ALP staining kit (Beyotime, Shanghai, China) following the manufacturer's instructions.

### RNA-sequencing (RNA-Seq) and data processing

At each time point, three replicate samples were prepared for RNA-Seq (4 time points, 12 samples in total). The total RNA of each sample was isolated as previous study reported [14]. Illumina HiSeq X Ten whole-genome expression arrays were used for RNA-Seq. Fastqc was conducted to perform the data quality control. Quantitative mapping was performed using fanse2 algorithm (parameter: -E 5%, -I 0, -S 13) with Rat_rn6_refMrnaas library. The differentially expressed genes (DEGs) between day 0 and day 7, 14 or 21 were analyzed with the limma package in R. The thresholds were *p* value < 0.05 and |log_2_Fold Change (FC)|≥ 1. DEGs were inputted to the web tool Venn Diagrams (http://bioinformatics.psb.ugent.be/webtools/Venn) for Venn analysis.

### Gene ontology (GO) enrichment analysis

The GO project provides a comprehensive source for functional genomics [15]. Database for Annotation, Visualization, and Integrated Discovery (DAVID; https://david.ncifcrf.gov/) was used for GO analysis to identify the characteristic biological function of DEGs. A few top terms (ranked by count) were selected for visualization.

### Gene set enrichment analysis (GSEA)

GSEA focuses on the gene set rather than the analysis of high-score genes. GSEA was performed with the OmicStudio tools, and the gene set was c2.cp.kegg.v7.4. The GSEA Bubble chart was plotted on https://www.bioinformatics.com.cn, a free online platform for data analysis and visualization. DEGs with *p* < 0.05 at three induction periods were used for GSEA analysis. The thresholds were set at |NES|> 1, pValue < 0.05 and qValue < 0.25.

### Construction of protein–protein interaction (PPI) networks

To assess the PPI network, Search Tool for the Retrieval of Interacting Genes (STRING) was used. Interaction scores among DEGs greater than 0.4 were considered significant, and the PPI network was constructed by Cytoscape software based on the massage of STRING. The Hub genes of the DEGs were screened by maximal clique centrality (MCC) of cytoHubba.

### Quantitative real-time polymerase chain reaction (qRT-PCR) analysis

*IL6*, *LIF*, *CSF3*, RUNX family transcription factor 2 (*RUNX2*), *BMP2*, bone gamma-carboxyglutamate protein (*BGLAP*), collagen type I alpha 1 chain (*COL1A1*), *ACKR3, VEGFD* and *ALPL* expression levels were detected by qRT-PCR. The primer sequences applied for the qRT‐PCR are summarized in Table 1. A Hipure Total RNA Mini kit (Magen, China) was used for the extraction of mRNA. And the standard qRT‐PCR experiment was performed according to our previous study [16]. Glyceraldehyde‐3‐phosphate dehydrogenase (*GAPDH*) was utilized as an internal reference.

**Table 1:** Primers sequence used for qRT-PCR

Gene names	Forward primer	Reverse primer
*IL6*	GCACTGGCGGAAGTCAATCT	TCTCAGCAGGCAGGTCTCAT
*LIF*	TCATGAGCCAGATCAGGAGC	CACAGCTTGTCCAGGTTGTTG
*CSF3*	TGAGCCAACTCCACAGCG	GCAAAGTCGGCGACATCC
*RUNX2*	GATGACGTCCCCGTCCATTC	TCTGAAGCACCTGAAATGCG
*BMP2*	AGACGACAGCGGTTTCCATC	CAAGTGGGTCACTTCCACCA
*COL1A1*	CTCCCAGAACATCACCTACCA	CAAACGTCGAAGCCGAAT
*BGLAP*	CCTCACTCTTGTCGCCCTGCTG	ACCACCTCGCTGCCCTCCCT
*ACKR3*	CCGAGCACAGCATCAAGG	TGGCGAGCAGGAAGTAGA
*VEGFD*	ACGCACGCTGAGGACTGG	CCTGGTGGACCGATGAGA
*ALPL*	AACCGCACTGAACTCCTG	GCTCGTACTGCATGTCCC
*GAPDH*	GTCATCATCTCAGCCCCCTC	GGATGCGTTGCTGACAATCT

#### Immunofluorescence staining

After 7 days of induction, cells were fixed with 4% paraformaldehyde and followed by incubating with 3% of H_2_O_2_ for 10 min at 25 ℃ to the block endogenous peroxidase activity. After blocking with normal goat serum, primary antibodies against IL6 (1:100, Affinity Biosciences, USA), STAT3 (1:100; Cell Signaling, USA), phosphorylation-STAT3 (p-STAT3; 1:100; Cell signaling, USA) and BGLAP (1:100; Proteintech, Wuhan, China) were used for incubation at 4 ℃ overnight. Then, the fluorescent dye-conjugated secondary antibodies (1:100; Bioss, Beijing, China) and 4′, 6‐diamidino‐2‐phenylindole (DAPI; Beyotime Biotechnology, Shanghai, China) were used for binding and nuclei staining. Images were captured with laser scanning confocal microscope (Leica, Germany), and the fluorescence intensity was semi-quantitatively analyzed by image J.

#### Western blotting (WB)

After the cells of each group were cultured for seven days, the total protein was extracted with PIPA. The protein concentration was quantified with the BCA kit (Beyotime Biotechnology, Shanghai, China), and the protein loading volume was adjusted to 70 μg for each group. After the end of gel running in 10% SDS-gel electrophoresis, the membranes were transferred and blocked with 5% skim milk blocking solution. Following blocking, the membranes were washed three times with TBST for 5 min each, and then, the primary antibodies were incubated overnight at low temperature. Primary antibodies were diluted at the following concentrations: STAT3 (1:1000; Cell Signaling, USA) and phosphorylation-STAT3 (p-STAT3; 1:1000; Cell signaling, USA). The secondary antibody was incubated for 1 h on the second day, and images were obtained in an Odyssey infrared imaging system.

#### Statistical analysis

The data were analyzed by one-way analysis of variance (ANOVA) using SPSS 22.0 software and exhibited as means ± standard deviation (SD). *p* < 0.05 was considered to be statistically significant.

## Results

### Osteogenic induction of BMSCs

**Fig. 1:**
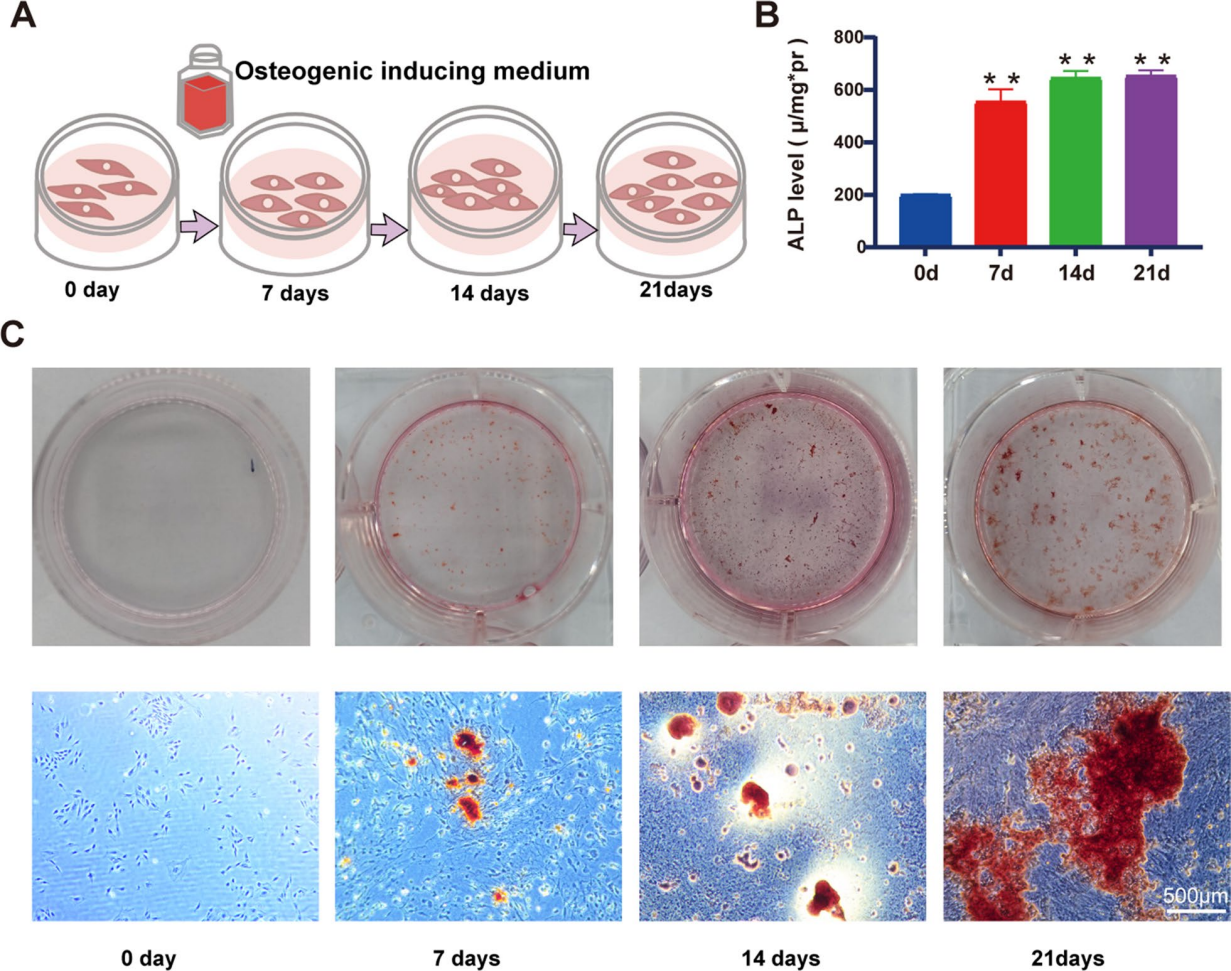
Validation osteogenic induction. **A** Induction period model of osteogenesis. **B** Alkaline phosphatase activity (ALP) assay (means ± SD, *n* = 3; **p < 0.01). **C** Alizarin red staining (Scale bar 500 μm)

BMSCs were induced with osteogenic induction medium to construct the osteogenic differentiation model (Fig. 1A). The efficiency of osteogenic differentiation of BMSCs at day 0, 7, 14 and 21 was verified by ALP activity assay (Fig. 1B) and alizarin red staining (Fig. 1C). The result showed that ALP activity and matrix mineralization induced by osteogenic induction medium were upregulated with the increase in inductive periods. It indicated that BMSCs were gradually differentiated toward osteoblast.

### Patterns of transcriptomics changes from BMSCs to osteoblast

**Fig. 2:**
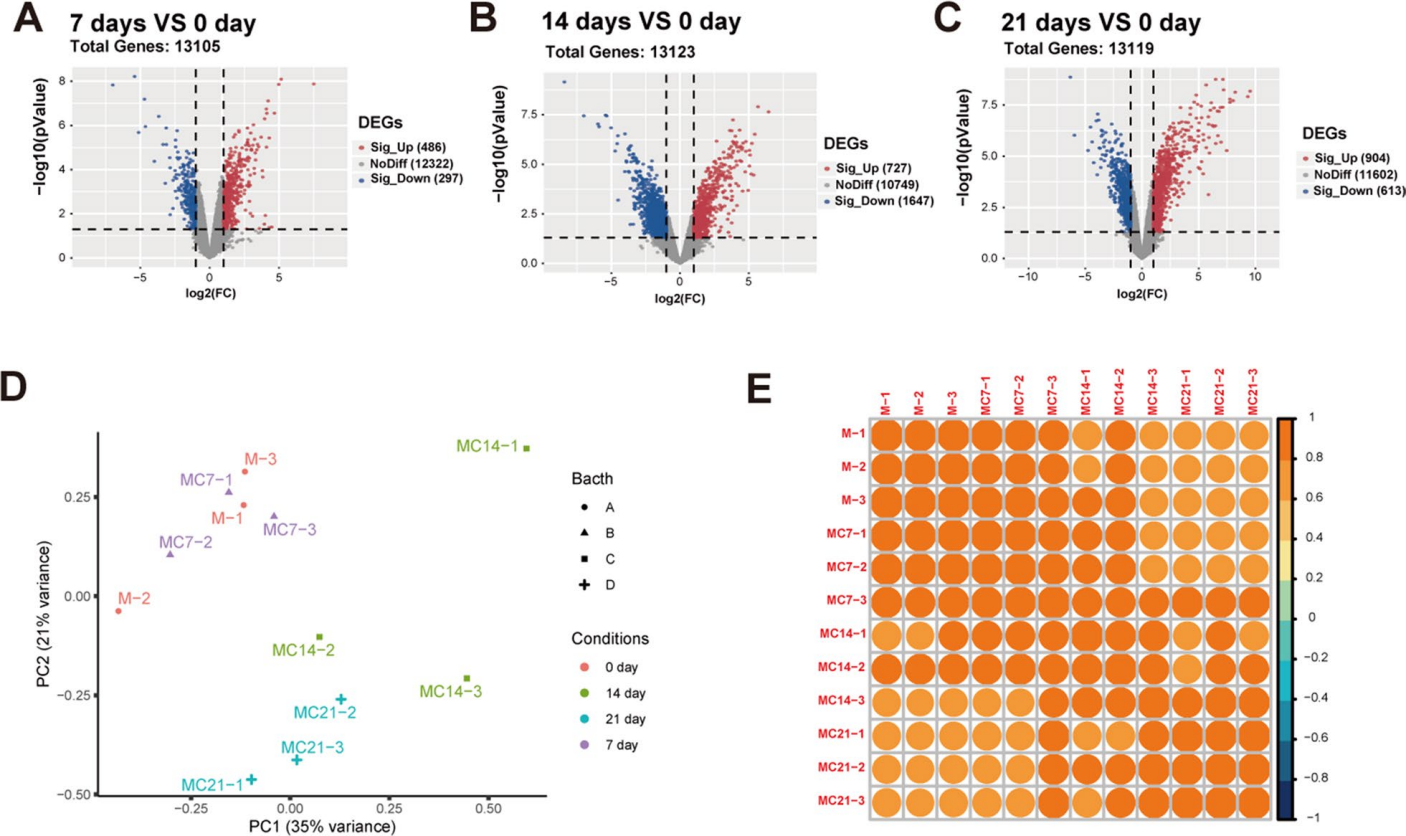
Identification of DEGs and sample correlation analysis. **A**–**C** Volcano plot for DEGs at day 7, 14 and 21 compared to day 0 (Red dots indicate significantly up-regulated genes. Blue dots indicate significantly down-regulated genes. Gray indicates no significantly difference. Differential gene screening conditions were *p* < 0.05 and |log_2_FC|≥ 1). **D** PCA results of samples’ expression profile. **E** Correlation analysis of differential gene expression at four-time points. The yellow color indicates positive correlation and the blue color indicates negative correlation. The darker color indicates higher correlation

To explore temporal transcriptome changes during osteogenic induction, the DEGs between day 7, 14, 21 and day 0, respectively, were analyzed. Compared to day 0, the down-regulated and up-regulated DEGs identified at day 7 were 297 and 486 (Fig. 2A). As shown in Fig. 2B, down-regulated and up-regulated DEGs between day 14 and 0 were 1647 and 727. In the comparison of day 21 and day 0, it demonstrated that 613 down-regulated and 904 up-regulated genes were recognized (Fig. 2C). Principal component analysis (PCA) was carried out by the expression profile, and four distinct groups which corresponded to different transition stages of osteoblast differentiation could be seen on the PCA diagram. As shown in Fig. 2D, PCA highlighted a clear distinction between samples from day 0–7 and day 14–21. Pearson correlation analysis also indicated compared with 7 days, the difference between the last two induction time points and day 0 was much distinct (Fig. 2E). Obviously, day 7 might be the cut-off point for early and late osteogenic differentiation from the transcriptome level.

**Fig. 3:**
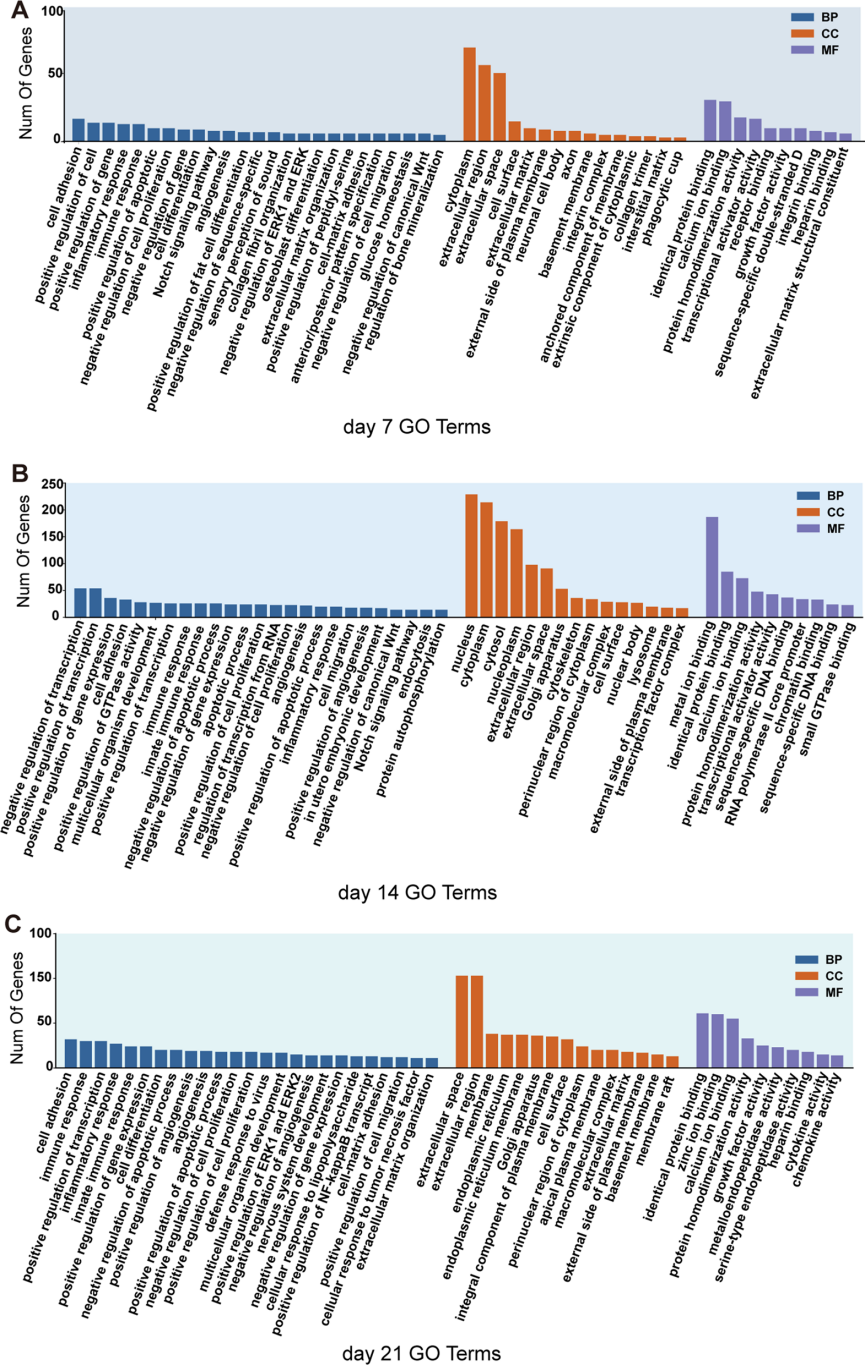
Functional enrichment analysis of DEGs. **A**–**C** GO enrich analysis results of the DEGs at day 7 (**A**), day 14 (**B**), and day 21 (**C**)

GO analysis was used to show which biological terms the DEGs were mainly enriched in, including biological process (BP), cellular component (CC) and molecular function categories (MF). Inflammatory response, immune response and angiogenesis were all enriched in the top 25 BP terms at day 7, 14 and 21 (Fig. 3A–C). In CC terms, cytoplasm, nucleus and extracellular space were the mainly terms at day 7, 14 and 21, respectively. In the MF terms, protein binding and ion binding were the mainly terms in at day 7, 14 and 21(Fig. 3A–C).

### Signaling pathway analysis and patterns of gene expression changes during osteogenesis

**Fig. 4:**
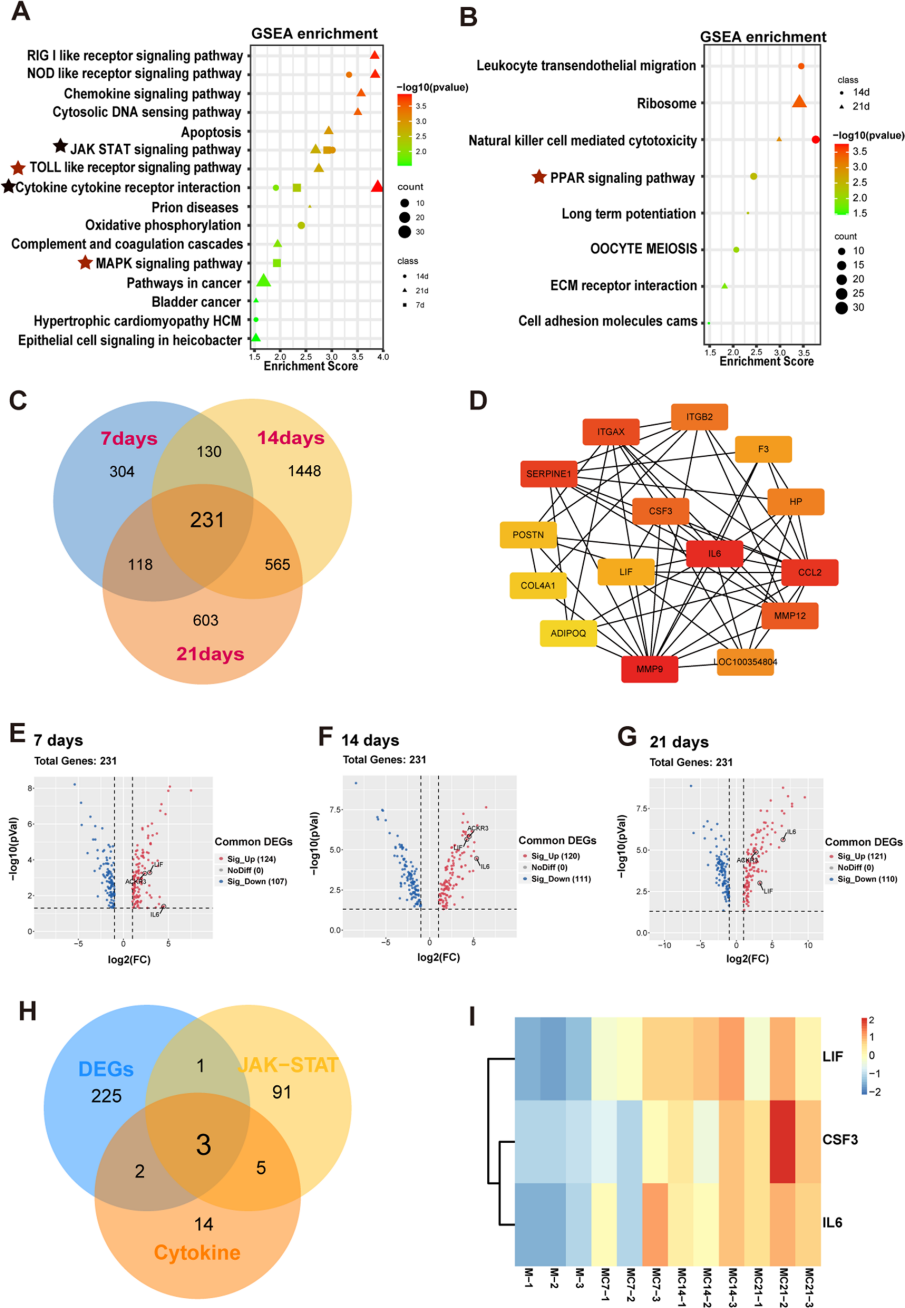
GSEA pathway and DEGs profiles analysis. **A** GSEA analysis of the DEGs of 7, 14 and 21 days, shown by bubble plot together. (NES > 1) **B** GSEA analysis of the DEGs of 14 and 21 days, shown by bubble plot together. (NES < -1) **C** The intersection of DEGs between 7, 14 and 21 days. **D** PPI network for intersected DEGs between 7, 14 and 21 days. **E**–**G** Volcano plot for intersected DEGs between 7, 14 and 21 days (Red dots indicate significantly up-regulated genes. Blue dots indicate significantly down-regulated genes). **H** Venn diagram shows the intersection of the 231 DEGs, JAK-STAT and cytokine–cytokine receptor interaction signaling pathways related genes. **I** Heatmap to show the common DEGs of the Venn diagram above

The enrichment analysis of signaling pathways using GSEA for DEGs showed that MAPK signaling pathway was active in the transcriptome of day 7, while PPAR signaling pathway was associated with down-regulated genes in the genome of day 14. In addition, Toll-like receptor signaling pathway was more active in the transcriptome of day 21. It also indicated that the cytokine–cytokine receptor interaction and JAK-STAT signaling pathways were involved in the osteogenic induction of BMSCs for 7, 14 and 21 days (Fig. 4A, B). Venn analysis was used to choose the common DEGs in the induction of 7, 14 and 21 days to identify the changes in differential genes that are stably expressed throughout osteogenic induction. A total of 231 common DEGs in each time points were discovered (Fig. 4C). Fifteen hub genes including IL6, LIF, CSF3 and etc. were found by PPI network (Fig. 4D). As shown in Volcano Plots of 231 EDGs in 7, 14 and 21 days, cytokines (IL6 and LIF) and marker of angiogenesis (ACKR3) were up-regulated (Fig. 4E–G). In addition, *IL6*, *LIF* and *CSF3* were the common genes overlapped with cytokine-cytokine receptor interaction signaling pathway, JAK-STAT signaling pathway and the 231 DEGs (Fig. 4H), and all of them were significantly upregulated (Fig. 4I). It suggested these three cytokines might promote the differentiation of BMSCs into osteoblasts by influencing the JAK-STAT signaling pathway. This result showed that during the process of osteogenic differentiation, the transcriptome would undergo dynamic changes, but the JAK-STAT signaling pathway would continue to activate. Therefore, we suspected that JAK-STAT signaling pathway was an osteogenic differentiation pathway that was less affected by induction time.

### Gene expression and signaling pathway verification

**Fig. 5:**
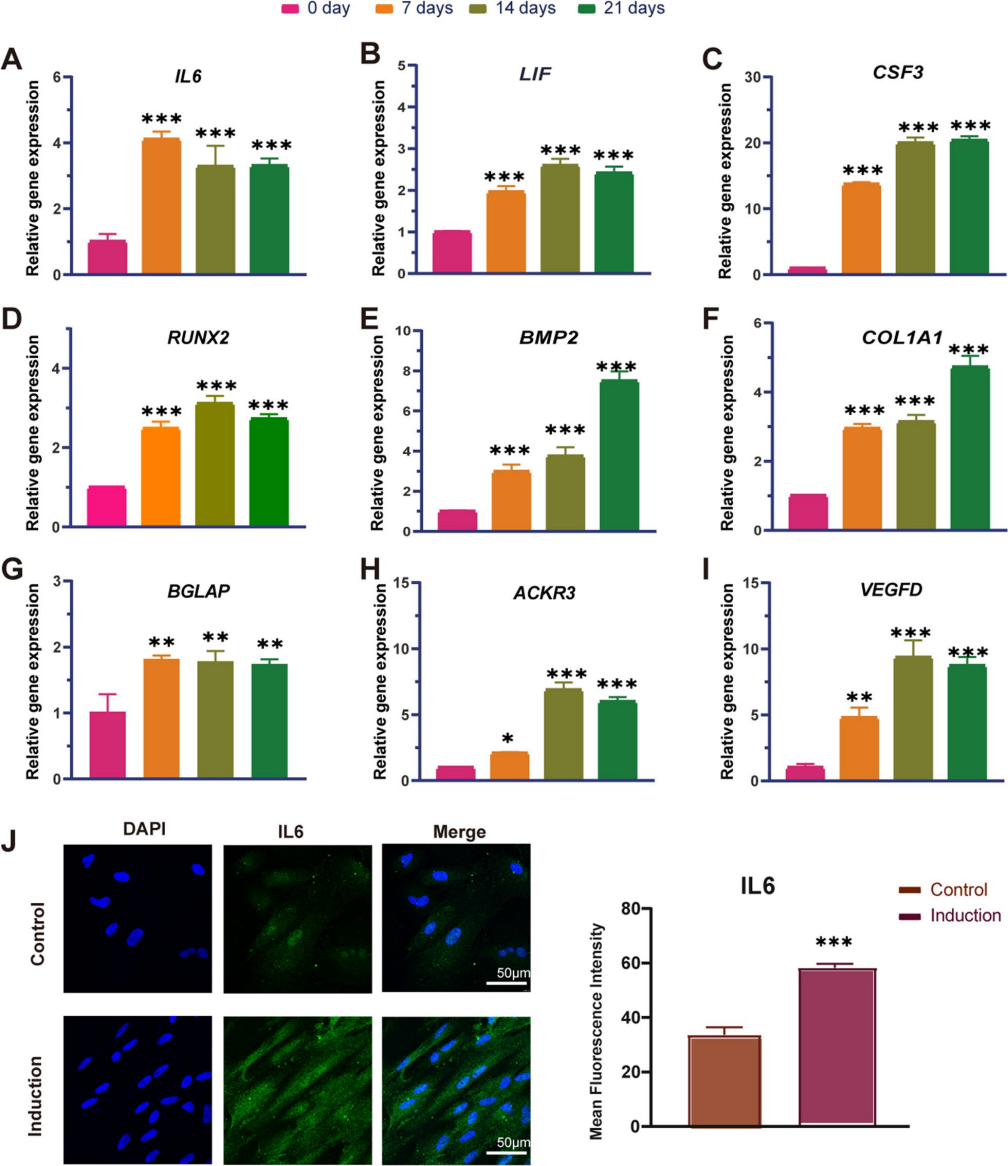
Gene expression verification by qRT-PCR and immunofluorescence staining. **A**–**I** The relative mRNA expression level of *IL6*, *LIF*, *CSF3*, *RUNX2, BMP2*, *COL1A1*, *BGLAP*, *ACKR3* and *VEGFD* after 0, 7, 14 and 21 days of inducing (means ± SD, *n* = 3; *p < 0.05, **p < 0.01, ***p < 0.001). **J** Immunofluorescence staining for IL6 in BMSCs cultured with osteogenic inducing medium for 7 days. Scale bars = 50 μm

In order to verify the reliability of the expression profiles of RNA-sequence, the gene expression levels in Cytokine/JAK/STAT3 signaling pathway, including cytokines (*IL6*, *LIF* and *CSF3*), osteogenic genes (*RUNX2, BMP2*, *COL1A1*, *BGLAP*) and angiogenic genes (*ACKR3* and *VEGF*), were further conformed by qRT-PCR. The result demonstrated that the expression levels of these genes were significantly increased by osteogenic induction, which were consistent with results of RNA-sequence (Fig. 5A–I).

**Fig. 6:**
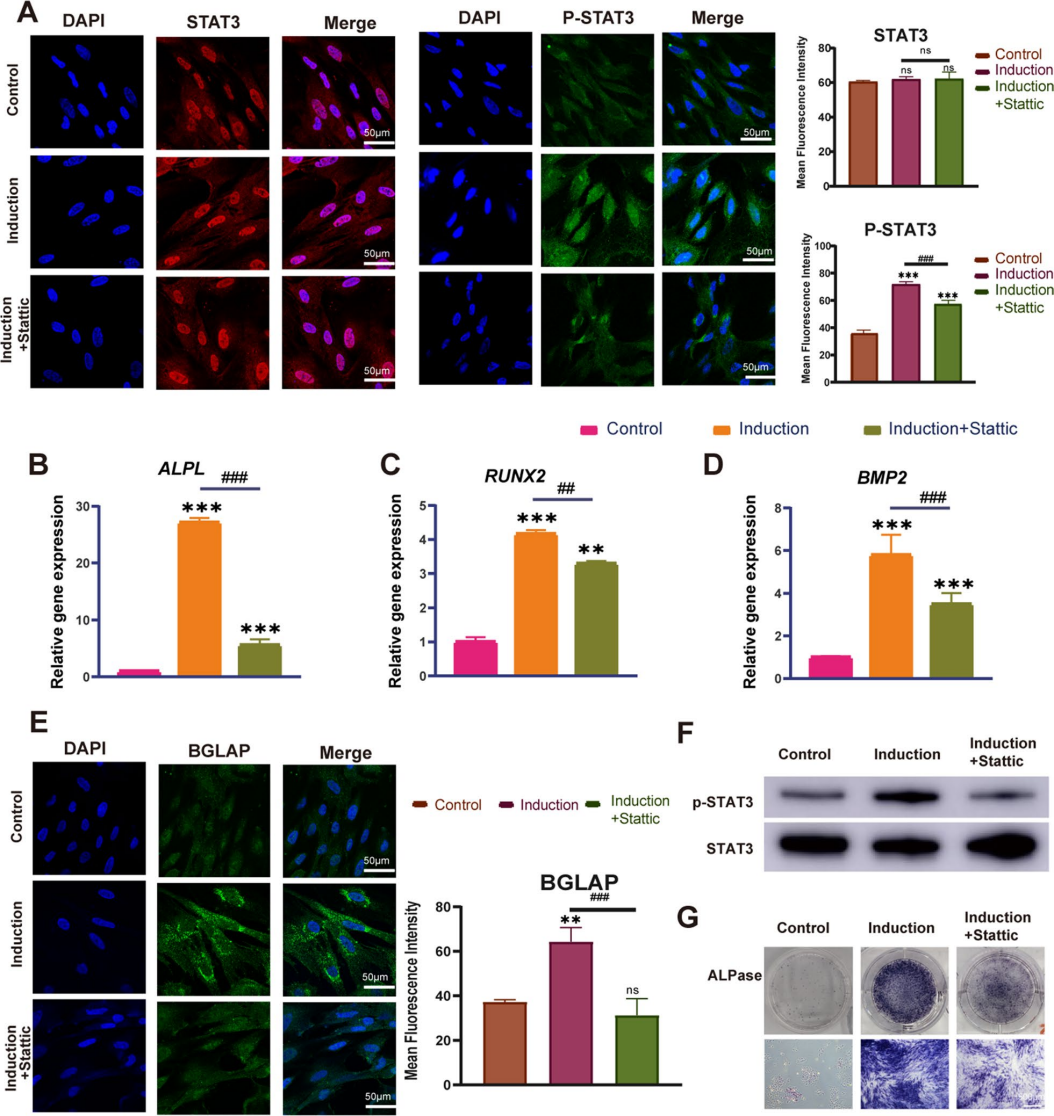
Role of JAK/STAT3 pathway in osteogenesis. **A** Immunofluorescence staining for STAT3 and p-STAT3 in BMSCs cultured with osteogenic inducing medium with or without Stattic for 7 days. **B**–**D** The relative mRNA expression levels of *ALPL*, *RUNX2*, *BMP2* measured by qRT-PCR. **E** Immunofluorescence staining for BGLAP. **F** The protein expression of P-STAT3 and STAT3 in BMSCs cultured with osteogenic inducing medium with or without Stattic for 7 days was detected by WB. **G** ALP staining (Scale bars = 500 μm). Mean ± SD, *n* = 3; *p < 0.05, **p < 0.01, ***p < 0.001, ^##^*p* < 0.01, ^###^*p* < 0.001

To further verify the mechanism of the osteogenesis of BMSCs induced by osteogenic induction medium, the protein expression levels of IL6, STAT3, phosphorylation of STAT3 and BGLAP were detected by immunofluorescence. After 7 days of induction, the fluorescence of IL6 was significantly upregulated (Fig. 5J), indicating IL6 was involved in the osteogenic induction process. The induction cooperated with or without Stattic (an inhibitor of STAT3 phosphorylation); no significant change was observed in expression level of STAT3 (Fig. 6A, F). The fluorescence of phosphorylation-STAT3 was significantly upregulated in the induction group, while decreased in addition of Stattic (Fig. 6A, F). The expression profile of osteogenic genes including *ALPL*, *RUNX2* and *BMP2* (Fig. 6B–D) and proteins including BGLAP (Fig. 6E) and ALP (Fig. 6G) was in consistent with phosphorylation-STAT3.

## Discussion

Therapy with BMSCs has been considered as a potential therapeutic strategy for many musculoskeletal disorders such as bone defects, osteogenesis imperfecta and osteoporosis [17]. However, the long-lasting underlying molecular mechanisms of osteogenic differentiation from BMSCs are still elusive. In the present study, we utilized the in vitro osteogenic induction model to study the temporal expression profiles in osteogenic differentiation of BMSCs. The crucial molecule mechanism that cytokines including IL6, LIF and CSF3 activated the phosphorylation of STAT3 to drive the osteogenesis and angiogenesis was identified by overlapping the common EDGs of three inductive periods.

The formation of hematoma at the defect site which induced by inflammation was a typical character of the primary phase of bone fracture healing [18]. Both macrophages and neutrophils were proved to influence the bone formation [19, 20]. In the osteogenic induction of BMSCs for 7, 14 and 21 days, inflammatory response and immune response were both contributed as the main biological process (Fig. 3). It was reported that stem cell differentiation to osteoblasts was a dynamic process. Initially, after seven days of induction, the MAPK pathway was activated, but after 14 days of induction, the PPAR pathway was predominant, showing that in the middle of induction, stem cells would further promote osteogenic differentiation by reducing lipogenic differentiation. After longer induction (21 days), the Toll pathway was gradually activated, while the pathways enriched to the first two time points were missing (Fig. 4A, B). All the above three pathways have been discussed in previous studies [21–23]. The correlation analysis also indicated the distinction between samples from four time-points (Fig. 2D–E). However, osteogenesis is occurred throughout the induction periods, so the co-expression EDGs were overlapped to explore the underlying molecular mechanisms. It demonstrated that the cytokine–cytokine receptor interaction and JAK-STAT signaling pathways were involved in all the osteogenic induction periods (Fig. 4A). The results of the PPI protein interaction analysis indicated the key role of IL6, LIF and CSF3 in the common DEGs (Fig. 4D). These inflammatory cytokines are involved in the Cytokine/JAK/SAT signaling pathway, and their expression levels were up-regulated in the induction of osteogenic induction medium (Figs. 4I, 5A–C). They might medicate the phosphorylation of STAT3 to modulate the osteogenesis of BMSCs. The osteogenic differentiation was inhibited by suppression the phosphorylation of STAT3 (Fig. 6). Increase in IL6 family cytokines activated the co-receptor gp130, as well as the JAK/STAT signaling pathway, resulting in the increased phosphorylation of STAT3 [24]. IL6 is known as an endogenous regulator in the innate immune system and the transformation from neutrophil to macrophage recruitment after injury [25]. It participates in the inflammatory phase of bone regeneration in various means. Overexpression of LIF promoted osteogenic differentiation of mouse renal tubular interstitial fibrosis by activating ERK and STAT3 pathway [26]. Different forms of mutations in colony-stimulating factor 3 receptor (CSF3R) were found to affect STAT3 activation [27]. JAK/STAT signaling pathway was also reported to participate osteogenic differentiation of MSCs stimulated by leptin and BMP9 [28].

Bone formation requires a well-coordinated combination of osteogenesis and angiogenesis [29]. BMSCs differentiated into osteoblasts accompanied with regeneration of neovasculature or neoangiogenesis which were also validated in this study (Fig. 5D–I). The angiogenesis in the newly formed bone was mediated by various cytokines and signaling pathways. CSF3 had been reported as a central downstream mechanism for the proangiogenic effects of TGF transforming growth factor β1 (TGFβ1) [8]. As evidenced by microvessel sprouting in aortic ring cultures, LIF overexpression MSCs promoted tube formation of endothelial cells in vitro [30]. Pro-inflammatory IL6 is one of the cytokines that stimulate angiogenic gene VEGF expression [31]. Ca^2+^/calmodulin-dependent protein kinase II (CaMKII) induced angiogenesis reliance on the regulation of interleukin-6 (IL6)/JAK/STAT3 signaling axis [32]. Here, we identified the cytokines (CSF3, IL6 and LIF) that contributed to osteogenesis and angiogenesis from the transcriptome perspective (Fig. 4D–I). These reports supported cytokines-mediated immuno-inflammation via activation of JAK-STAT3 signaling pathway as a promoter of angiogenesis and osteogenesis. Last but not the least, this research seemed to be the first time to reveal the temporal transcriptome of rabbit BMSCs differentiating into osteoblasts, which provides valuable reference for solving the differences between species.

## Conclusions

In summary, the study comprehensively investigated the transcriptomic profiles of osteogenesis from BMSCs, as well as the angiogenesis profile in the process of osteogenic differentiation. In addition, crucial pro-osteogenesis regulators including IL6, LIF and CSF3, and JAK/STAT3 signaling pathway that were positivity associated with angiogenesis and osteogenesis were identified. This work might give a new insight in understanding the molecular regulation of long-lasting osteogenic differentiation.
